# Reparo de aneurisma de artéria ilíaca roto em criança

**DOI:** 10.1590/1677-5449.008616

**Published:** 2017

**Authors:** Fernando Massaru Hoshiko, Elisa Helena Subtil Zampieri, Marcelo Bellini Dalio, Nei Rodrigues Alves Dezotti, Edwaldo Edner Joviliano

**Affiliations:** 1 Universidade de São Paulo – USP, Faculdade de Medicina de Ribeirão Preto – FMRP, Departamento de Cirurgia e Anatomia, Divisão de Cirurgia Vascular e Endovascular, Ribeirão Preto, SP, Brasil.

**Keywords:** aneurisma ilíaco, aneurisma roto, criança, enxerto vascular

## Abstract

Relatamos o caso de uma menina de 12 anos que deu entrada na unidade de emergência com quadro de abdome agudo hemorrágico, massa abdominal pulsátil e instabilidade hemodinâmica. Confirmado o diagnóstico de aneurisma roto de artéria ilíaca direita, foi realizada correção cirúrgica de emergência por reparo aberto com reconstrução extra-anatômica, utilizando enxerto sintético de fino calibre, compatível com a anatomia. O tratamento foi bem-sucedido e a criança apresentou evolução favorável em curto prazo.

## INTRODUÇÃO

Aneurismas de artéria ilíaca (AAIs) isolados são raros na população geral. Sua prevalência é estimada em 0,03% em relatos de autópsia. A ocorrência de aneurismas arteriais em crianças é ainda mais rara. Por ser raro e de difícil palpação, o diagnóstico de AAI é comumente tardio, quando geralmente apresenta elevadas dimensões. Nessa situação, o AAI apresenta alto risco de rotura e alta taxa de mortalidade^1^.

Neste relato, descrevemos o caso de uma criança de 12 anos de idade com AAI roto. Foram realizadas correção cirúrgica por reparo aberto e reconstrução extra-anatômica com enxerto de politetrafluoroetileno (PTFE). Essa técnica de reconstrução vascular não é usualmente empregada e foi escolhida devido à situação de emergência, à anatomia e à disponibilidade de material para enxerto. O tratamento foi bem-sucedido, e a criança apresentou evolução favorável em curto prazo.

### Parte I – Situação clínica

Paciente do sexo feminino, 12 anos de idade, deu entrada na unidade de emergência com quadro de 12 horas de dor abdominal irradiada para o membro inferior direito. Negava febre durante o período. Ao exame físico, apresentava-se descorada, taquicárdica e hipotensa (choque hipovolêmico grau II). À palpação abdominal, apresentava dor difusa, com sinais de peritonismo e massa pulsátil, expansível e dolorosa em hipogástrio e fossa ilíaca direita. Trazia um resultado de exame laboratorial realizado na atenção primária com valor de hemoglobina de 10,5 mg/dL, que foi repetido na admissão, quando se constatou valor de 8,1 mg/dL. Os demais exames laboratoriais não apresentavam alterações.

**Figura 1:**
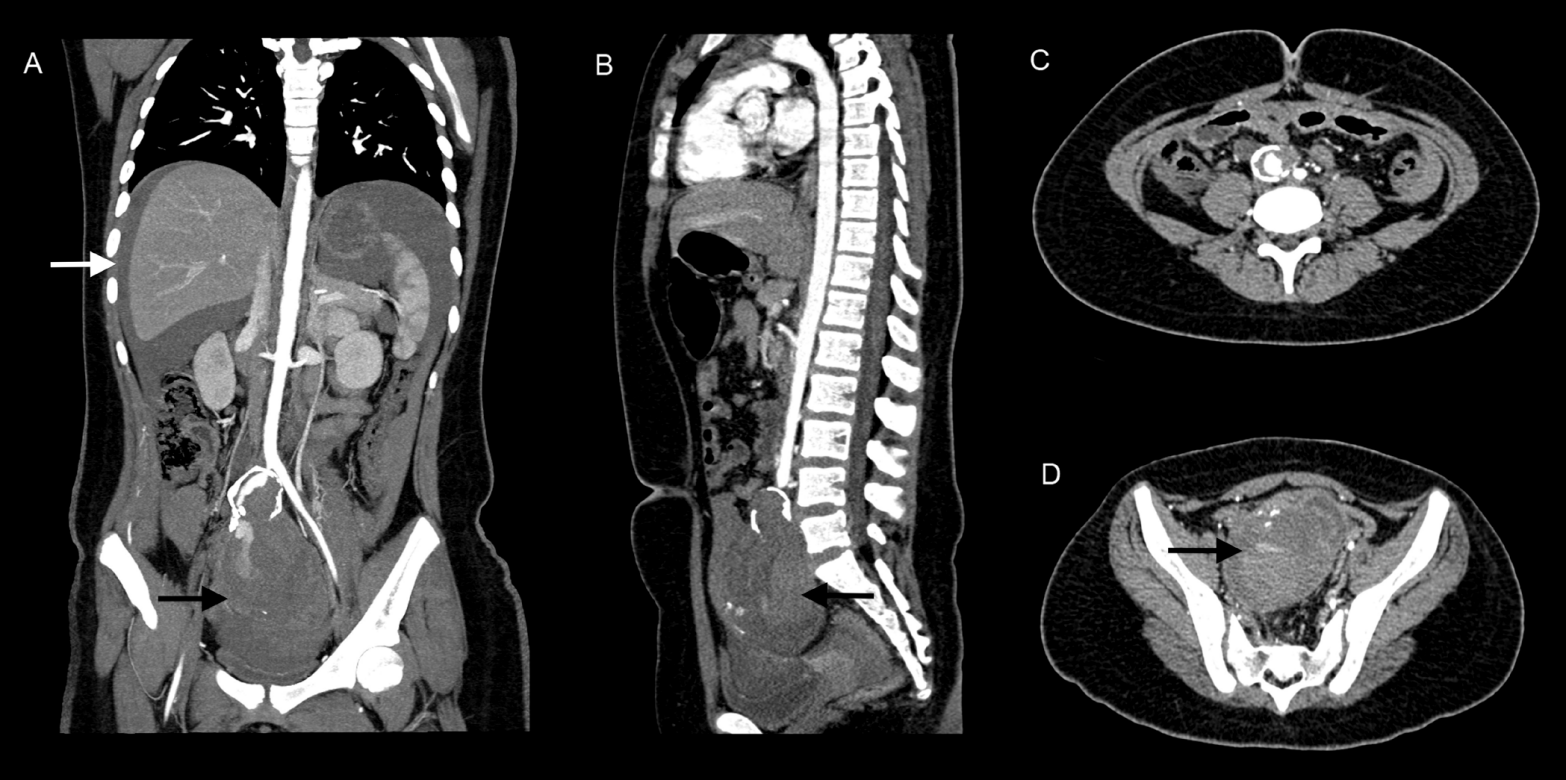
Angiotomografia computadorizada com contraste endovenoso (fase arterial) em cortes coronal (A), sagital (B) e axiais (C e D) evidenciando presença de sangue na cavidade peritoneal (seta branca) e aneurisma de artéria ilíaca comum direita com sinais de rotura (seta preta).

Devido a uma forte suspeita de abdome agudo vascular hemorrágico, foi providenciada a reposição volêmica por meio de dois acessos venosos periféricos calibrosos e infusão de cristaloides e concentrados de hemácias. Como a paciente manteve níveis pressóricos estáveis, foi encaminhada a angiotomografia computadorizada (angioTC) de aorta total e vasos ilíacos, que evidenciou um aneurisma fusiforme acometendo a artéria ilíaca comum direita, medindo 9,3 cm em seu maior diâmetro. Havia sinais de rotura e moderada quantidade de sangue na cavidade peritonial (Figura 1). A paciente foi prontamente encaminhada à cirurgia de emergência. Como se tratava de uma criança pré-púbere com dimensões reduzidas das artérias aorta, ilíacas e femorais, optamos por executar o reparo aberto do aneurisma em vez do tratamento endovascular. Sob anestesia geral, a paciente foi submetida a laparotomia mediana xifopúbica e acesso transperitonial para aorta e vasos ilíacos. A aorta tinha 6 mm de diâmetro em sua porção infrarrenal. Observamos um volumoso aneurisma na artéria ilíaca comum direita. A massa aneurismática e o hematoma circundante ocupavam todo o diminuto espaço da pequena bacia, impedindo a dissecção para controle distal. A criança apresentava, naquele momento, instabilidade hemodinâmica. Além disso, uma prótese bifurcada com dimensões compatíveis com a aorta da criança não estava disponível no momento.

### Parte II – O que foi feito

Foi realizado clampeamento de aorta infrarrenal para controle proximal. Como o acesso à cavidade pélvica era restrito, optamos por controlar as artérias femorais comuns por meio de inguinotomia longitudinal bilateral. O saco aneurismático foi então aberto, os trombos murais foram removidos, e o sangramento por refluxo nas artérias ilíaca comum esquerda, ilíaca interna direita e lombares foi controlado com sutura dos óstios. A reconstrução vascular foi realizada com prótese tubular de PTFE 5 mm (Maquet®, Wayne, Nova Jérsei, EUA), que era compatível com o diâmetro da aorta abdominal. Foi realizada anastomose terminoterminal de aorta-prótese em segmento infrarrenal e anastomose terminolateral de prótese-artéria femoral comum direita. Devido a hematoma extenso da pelve, anatomia distorcida e processo inflamatório local, a dissecção correta dos vasos e o pinçamento da artéria ilíaca comum esquerda não foram possíveis. Optamos pelo controle urgente da hemostasia e pela realização de derivação femorofemoral cruzada da direita para a esquerda, utilizando o mesmo enxerto de PTFE 5 mm. A anastomose direita foi de prótese-prótese lateroterminal e a esquerda foi de prótese-artéria femoral comum esquerda terminolateral. Dessa forma, também preservamos a artéria ilíaca comum esquerda sadia para uma futura intervenção. A paciente necessitou de transfusão de nove concentrados de hemácias, 400 mL de plasma e dois concentrados de plaquetas.

Após o procedimento, a paciente foi mantida em unidade de terapia intensiva (UTI), intubada, sedada e em uso de aminas vasoativas. Foi extubada no segundo dia após o procedimento e recebeu alta da UTI no terceiro dia. A paciente apresentava as extremidades bem perfundidas com pulsos podálicos palpáveis. Recebeu alta hospitalar no sexto dia pós-operatório.

**Figura 2:**
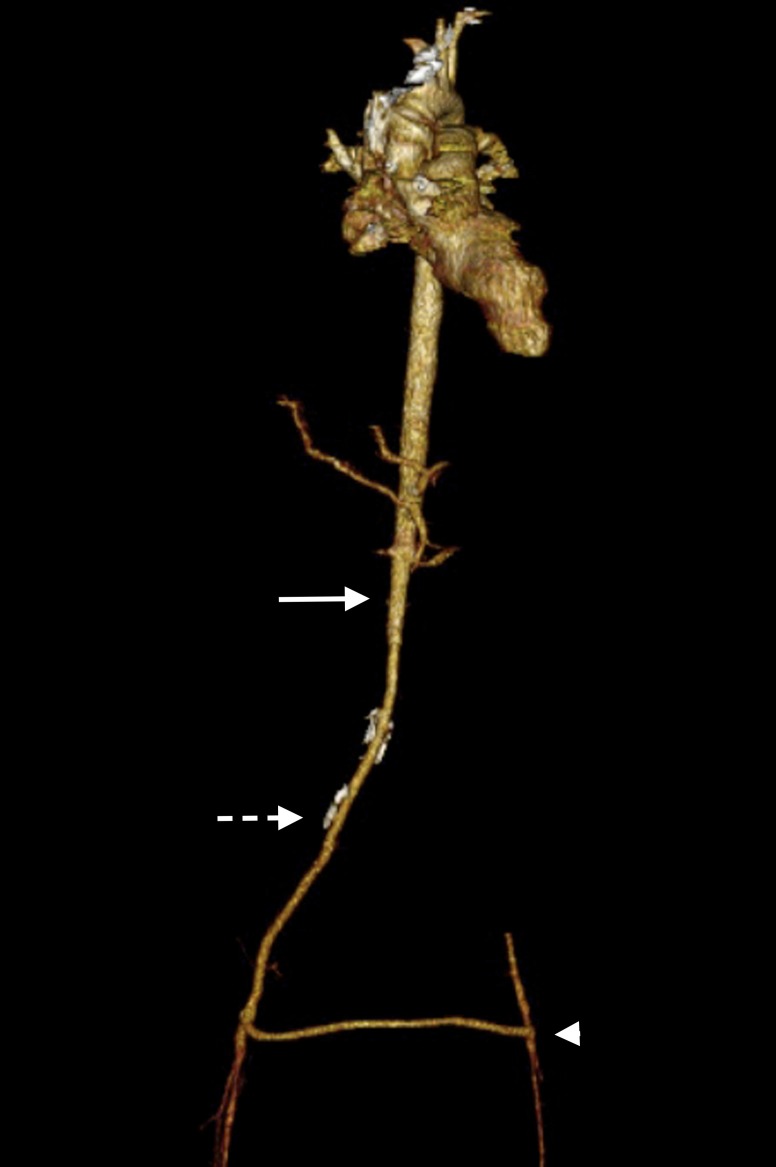
Angiotomografia computadorizada com contraste endovenoso (fase arterial) em reconstrução tridimensional do pós-operatório evidenciando a aorta (seta contínua), enxerto aortofemoral direito (seta pontilhada) e *bypass* femorofemoral cruzado (cabeça de seta).

Na consulta de retorno, 40 dias após o procedimento, a paciente negou queixas abdominais ou claudicação intermitente. Os pulsos podálicos eram palpáveis. A angioTC de controle mostrou perviedade da prótese aortofemoral direita e da derivação femorofemoral cruzada (Figura 2).

## DISCUSSÃO

A doença aneurismática em crianças é um evento raro. A maior série descrita na literatura foi publicada em 1991 por Sarkar et al.^2^, que relataram 165 casos. O AAI isolado é ainda mais raro, representando 0,1% de todos os casos. No entanto, apresenta alto risco de rotura e taxa de mortalidade de 50-75%^1^. Neste relato, descrevemos um caso de aneurisma de ilíaca roto em criança. Trata-se de uma situação rara e desafiadora para o cirurgião vascular, que deve realizar o rápido diagnóstico e instituir o tratamento cirúrgico emergencial.

Evidências clinicopatológicas de aneurismas arteriais em crianças são raras. No entanto, com o objetivo de direcionar a investigação diagnóstica e as possibilidades terapêuticas, foram relatadas na literatura nove situações que podem estar envolvidas no desenvolvimento de aneurismas: (1) aneurismas infecciosos, (2) aortoarterite por células gigantes, (3) doenças autoimunes do tecido conectivo, (4) doença de Kawasaki, (5) degeneração da média nas síndromes de Marfan e Ehlers-Danlos, (6) outras causas de degeneração da média não inflamatórias (esclerose tuberosa), (7) displasia vascular, (8) doenças congênitas e (9) pseudoaneurismas^2^. Para essa investigação, é relevante também pesquisar antecedente familiar de aneurisma, doenças infecciosas crônicas, presença de febre associada ao desenvolvimento do aneurisma, trauma abdominal importante prévio e antecedente de doença do tecido conectivo. Além disso, a coleta de material da parede do aneurisma para cultura e exame anatomopatológico pode esclarecer ou facilitar o entendimento da causa do desenvolvimento do aneurisma e orientar o tratamento pós-operatório.

Não há consenso com relação ao manejo de casos de AAI em crianças, pois sua ocorrência é muito rara. O tratamento de eleição ainda é o reparo aberto com interposição de enxerto. Não há relato de tratamento endovascular de AAI em crianças.

Em crianças, a correção dos aneurismas envolve considerações especiais: os vasos apresentam dimensões reduzidas, há uma expectativa de crescimento do paciente e a expectativa de vida é longa. Enxertos apropriados para essa situação ainda não estão disponíveis^3^. Chithra et al.^4^ e Zimmermann et al.^5^ utilizaram, em cirurgias eletivas, enxerto de veia femoral para o reparo aberto do aneurisma de ilíaca. Já Kaye et al. sugerem utilizar enxertos homólogos criopreservados em pacientes mais jovens e enxertos sintéticos em crianças mais velhas^3^.

No nosso caso, em uma cirurgia de emergência com o paciente instável, o uso de enxertos autólogos não era uma possibilidade, pois aumentaria drasticamente o tempo cirúrgico, a morbidade e a mortalidade. Como solução, foi utilizado um enxerto sintético. Não havia próteses bifurcadas disponíveis para as dimensões das artérias da paciente e, como única opção, utilizamos prótese reta PTFE de 5 mm (Maquet®, Wayne, Nova Jérsei, EUA). Com o objetivo de evitar o acesso à pelve e preservar a artéria ilíaca comum esquerda sadia para uma futura intervenção, optamos pelo enxerto extra-anatômico femorofemoral cruzado. Esse enxerto tem uma perviedade inferior em relação a reconstrução anatômica^6^. Utilizamos esse enxerto como opção provisória para retirar a paciente da situação crítica. Futuramente, após a paciente completar a fase de crescimento, planejamos reabordagem para reconstrução anatômica com prótese de tamanho adequado.

Por fim, também deve-se ter uma vigilância rigorosa em longo prazo desses pacientes para o desenvolvimento posterior de doenças do tecido conjuntivo ou aneurismas metacrônicos.

## CONCLUSÃO

O reparo aberto de aneurisma roto de artéria ilíaca em criança, utilizando prótese de PTFE, é factível e foi bem-sucedido em situação de emergência.
