# Supplementary material for: Reparo de aneurisma de artéria ilíaca roto em criança
Source: J Vasc Bras. 2017 Jan-Mar;16(1):48–51. [Article in Portuguese] doi: 10.1590/1677-5449.008616 (PMC5829692; doi:10.1590/1677-5449.008616)
Supplement: Translation

# Translation: Repair of ruptured iliac artery aneurysm in a child

**Authors:** Fernando Massaru Hoshiko, Elisa Helena Subtil Zampieri, Marcelo Bellini Dalio, Nei Rodrigues Alves Dezotti, Edwaldo Edner Joviliano

**Affiliations:** 1 Universidade de São Paulo – USP, Faculdade de Medicina de Ribeirão Preto – FMRP, Departamento de Cirurgia e Anatomia, Divisão de Cirurgia Vascular e Endovascular, Ribeirão Preto, SP, Brazil.

**Keywords:** ruptured aneurysm, iliac aneurysm, child, vascular graft

## Abstract

We describe the case of a 12-year-old girl who presented at the emergency department with hemorrhagic acute abdomen, an abdominal pulsating mass and hemodynamic instability. A diagnosis of ruptured right iliac artery aneurysm was confirmed and an emergency open repair procedure was performed with extra-anatomic reconstruction, using a small-caliber synthetic graft, compatible with her anatomy. The treatment was successful and the child was doing well at short-term follow-up.

## INTRODUCTION

Isolated iliac artery aneurysms (IAAs) are rare in the general population. Their prevalence is estimated at 0.03% in autopsy reports. Arterial aneurysms occurring in children are even rarer. Because of their rarity and the difficulty of palpation, IAAs are often diagnosed late, by which point they have generally attained large dimensions. In these situations an IAA has a high risk of rupture and an elevated mortality rate.^1^

In this report, we describe the case of a 12-year-old child with a ruptured IAA. Open surgical repair was performed with extra-anatomic reconstruction using a polytetrafluoroethylene (PTFE) graft. This vascular reconstruction technique is not usually employed and it was chosen because of the emergency situation, the patient’s anatomy, and the availability of material for grafting. The treatment was successful and the child was doing well at short-term follow-up.

### Part I – Clinical situation

A 12-year-old female patient was admitted to the emergency department suffering from abdominal pains, irradiating to the right lower limb, and with onset 12 hours previously. She had no fever during this period. On physical examination she was pale and had tachycardia and had low blood pressure (stage II hypovolemic shock). Abdominal palpation provoked diffuse pain and revealed signs of peritonitis and a pulsating, expanding, and painful mass in the hypogastric and right inguinal areas. She had brought the result of a laboratory test conducted by a primary care service, showing hemoglobin at 10.5 mg/dL, and the test was repeated on admission, at which point the level was 8.1 mg/dL. Other laboratory test results were unremarkable.

In view of the strong suspicion of vascular hemorrhagic acute abdomen, volume resuscitation was given via two large caliber peripheral venous accesses with infusion of crystalloid fluids and packed red blood cells. Since the patient was maintaining stable blood pressures, she was sent for computed tomography angiography (angioCT) of the full aorta and iliac vessels, which showed a fusiform aneurysm involving the right common iliac artery and measuring 9.3 cm at its largest diameter. There were signs of rupture and a moderate quantity of blood in the peritoneal cavity (Figure 1). The patient was promptly sent for emergency surgery. Since she was a prepubescent child with small aorta and iliac and femoral arteries, we decided to conduct an open repair of the aneurysm, rather than attempt endovascular repair. Under general anesthesia, a long midline laparotomy was performed and transperitoneal access obtained to the aorta and iliac vessels. The infrarenal portion of the aorta had a diameter of 6 mm. We observed a large-volume aneurysm of the right common iliac artery. The aneurysmal mass and surrounding hematoma occupied the entirety of the little space there was within the patient’s small pelvic cavity, preventing dissection for distal control. By this point, the child had become hemodynamically unstable. Additionally, a bifurcated prosthesis with dimensions compatible with the child’s aorta was not immediately available.

**Figure 1:** Computed tomography angiography with intravenous contrast (arterial phase), coronal (A), sagittal (B), and axial (C and D) slices, showing blood in the peritoneal cavity (white arrow) and an aneurysm of the right common iliac artery with signs of rupture (black arrow).

### Part II – What was done

The infrarenal aorta was clamped to achieve proximal control. Since there was restricted access to the pelvic cavity, we decided to control the common femoral arteries via bilateral longitudinal inguinotomy. The aneurysm sac was then opened, the mural thrombi were removed, and the reflux bleeding from the left common iliac, right internal iliac, and lumbar arteries was controlled by suturing their ostia. Vascular reconstruction was conducted with a 5 mm tubular PTFE prosthesis (Maquet®, Wayne, New Jersey, United States), which was compatible with the diameter of the abdominal aorta. An end-to-end anastomosis was constructed between the infrarenal segment of the aorta and the prosthesis and an end-to-side anastomosis between the prosthesis and the right common femoral artery. The extensive hematoma of the pelvis, distorted anatomy, and localized inflammatory process prevented correct dissection of the vessels and clamping of the left common iliac artery. We decided to accomplish urgently-needed hemostatic control by constructing a right-to-left femorofemoral crossover bypass, using the same 5 mm PTFE graft. The right-side anastomosis was end-to-side prosthesis-to-prosthesis while the left was an end-to-side anastomosis of the left common femoral artery to the prosthesis. We were thus able to preserve the left common iliac artery intact for future intervention. The patient required transfusion of nine units of packed red blood cells, 400 mL of plasma and two units of concentrated platelets.

After the procedure, the patient was transported to the intensive care unit (ICU), intubated, sedated, and administered vasoactive amines. She was extubated on the second day after the procedure and discharged from the ICU on the third day. She had well-perfused extremities with palpable pedal pulses. She was discharged from hospital on the sixth day after surgery.

At a follow-up consultation 40 days after the procedure, the patient said she was free from abdominal complaints and from intermittent claudication. Her pedal pulses were palpable. Control angioCT showed that the right aortofemoral prosthesis and the femorofemoral crossover bypass were patent (Figure 2).

**Figure 2:** Three-dimensional reconstruction of postoperative computed tomography angiography with intravenous contrast (arterial phase) showing the aorta (continuous arrow), right aortofemoral graft (dotted arrow) and femorofemoral crossover bypass (arrowhead).

## DISCUSSION

Aneurysmal disease is a rare event in children. The largest series described in the literature was published in 1991 by Sarkar et al.^2^ and comprised 165 cases. Isolated IAAs are rarer still, accounting for 0.1% of all cases. However, they have a high risk of rupture and the mortality rate is 50-75%.^1^ In this report we have described the case of a child with a ruptured iliac aneurysm. This is a rare situation and a challenge for the vascular surgeon, who must arrive at a diagnosis rapidly and provide emergency surgical treatment.

Clinicopathological evidence of arterial aneurysms in children is rare. However, with the objective of providing guidance for diagnostic investigations and treatment options, there follows a list of nine situations that are described in the literature as possibly involved in development of aneurysms: (1) arterial infection, (2) giant-cell arteritis of the aorta, (3) autoimmune connective tissue diseases, (4) Kawasaki disease, (5) medial degeneration in Marfan and Ehlers-Danlos syndromes, (6) other, non-inflammatory causes of degeneration of the tunica media (tuberous sclerosis), (7) vascular dysplasia, (8) congenital diseases and (9) pseudoaneurysms.^2^ Also of relevance to diagnostic investigation are family history of aneurysms, chronic infectious diseases, fever associated with development of the aneurysm, previous significant abdominal trauma, and history of connective tissue disease. Additionally, taking samples of material from the aneurysm wall for cultures and anatomopathological examination may elucidate or facilitate understanding of the causes of development of the aneurysm and provide guidance for postoperative treatment.

There is no consensus with relation the management of IAA cases in children, since it occurs very rarely. Elective treatment remains open repair with interposition of a graft. There are no reports of endovascular treatment of IAAs in children.

Repair of aneurysms in children involves specific considerations: the vessels have smaller dimensions, there is the expectation that the patient will grow, and life expectancy is long. Grafts that are appropriate for this situation are not yet available.^3^ Chithra et al.^4^ and Zimmermann et al.^5^ conducted elective surgeries using femoral vein grafts to conduct open repairs of iliac aneurysms. Kaye et al. suggested using cryopreserved homologous grafts in younger patients and synthetic grafts in older children.^3^

In our case, during emergency surgery on an unstable patient, autologous grafting was not an option, because it would have drastically increased the duration of the operation, increasing morbidity and raising the likelihood of mortality. Our solution was to use a synthetic graft. Bifurcated prostheses suitable for the dimensions of the patient’s arteries were not available and, since it was the only option, we used a straight 5mm PTFE prosthesis (Maquet®, Wayne, New Jersey, United States). In order to avoid accessing the pelvis and to preserve the left common iliac artery intact for future intervention, we decided to construct an extra-anatomic femorofemoral crossover graft. This graft offers reduced patency compared with anatomic reconstruction.^6^ We employed this graft as a provisional option to extricate the patient from the critical situation. In the future, after the patient has completed her growth phase, we plan to reoperate and conduct an anatomic reconstruction with a suitable-sized prosthesis.

Finally, these patients should also be subject to rigorous long-term surveillance to detect any later development of connective tissue diseases or metachronous aneurysms.

## CONCLUSIONS

Open repair of a ruptured iliac artery aneurysm in a child, using a PTFE prosthesis is achievable and in this case it was successful in an emergency situation.
